# Depression and assets during the COVID-19 pandemic: A longitudinal study of mental health across income and savings groups

**DOI:** 10.1371/journal.pone.0304549

**Published:** 2024-06-14

**Authors:** Catherine K. Ettman, Gregory H. Cohen, Salma M. Abdalla, C. Ross Hatton, Brian C. Castrucci, Rachel H. Bork, Sandro Galea

**Affiliations:** 1 Department of Health Policy and Management, Johns Hopkins Bloomberg School of Public Health, Baltimore, Maryland, United States of America; 2 Department of Epidemiology, Boston University School of Public Health, Boston, Massachusetts, United States of America; 3 deBeaumont Foundation, Bethesda, Maryland, United States of America; Western Health, AUSTRALIA

## Abstract

The prevalence of depression in U.S. adults during the COVID-19 pandemic has been high overall and particularly high among persons with fewer assets. Building on previous work on assets and mental health, we document the burden of depression in groups based on income and savings during the first two years of the COVID-19 pandemic. Using a nationally representative, longitudinal panel study of U.S. adults (N = 1,271) collected in April-May 2020 (T1), April-May 2021 (T2), and April-May 2022 (T3), we estimated the adjusted odds of reporting probable depression at any time during the COVID-19 pandemic with generalized estimating equations (GEE). We explored probable depression—defined as a score of ≥10 on the Patient Health Questionnaire-9 (PHQ-9)—by four asset groups, defined by median income (≥$65,000) and savings (≥$20,000) categories. The prevalence of probable depression was consistently high in Spring 2020, Spring 2021, and Spring 2022 with 27.9% of U.S. adults reporting probable depression in Spring 2022. We found that there were four distinct asset groups that experienced different depression trajectories over the COVID-19 pandemic. Low income-low savings asset groups had the highest level of probable depression across time, reporting 3.7 times the odds (95% CI: 2.6, 5.3) of probable depression at any time relative to high income-high savings asset groups. While probable depression stayed relatively stable across time for most groups, the low income-low savings group reported significantly higher levels of probable depression at T2, compared to T1, and the high income-low savings group reported significantly higher levels of probable depression at T3 than T1. The weighted average of probable depression across time was 42.9% for low income-low savings groups, 24.3% for high income-low savings groups, 19.4% for low income-high savings groups, and 14.0% for high income-high savings groups. Efforts to ameliorate both savings and income may be necessary to mitigate the mental health consequences of pandemics.

## Introduction

Depression is common in the U.S. [1]. In 2019, one in five U.S. adults reported symptoms of depression [2], which increased during the COVID-19 pandemic [3]. Depression is associated with other forms of mental illness [4, 5], physical illness [6–9], and even early mortality [10]. Depression is costly for individuals, families, and employers in terms of impaired functionality, treatment, and lost productivity [11–14]. Economic disparities in depression can lead to heightened inequities across health and other outcomes, further widening gaps between persons with and without resources.

Depression and psychological distress increased during the COVID-19 pandemic [15–18] and certain groups such as those with low income were more likely to experience poor mental health than others [19–21]. Mental health is the product of environments that shape health outcomes [22, 23]. These environments shifted significantly during the COVID-19 pandemic. Efforts to protect persons from contracting COVID-19 led to widescale economic disruption including business closure and job loss. The period that followed initial stay at home orders has been called “the most unequal recession in history” [24], with low income groups bearing the brunt of economic and employment loss in the face of COVID-19 restrictions. The groups more likely to have precarious financial situations leading into the COVID-19 pandemic were the very groups more likely to experience stressors during the pandemic; low asset groups reported the most disruption to and least control over their environments during the pandemic [25], leading to widening gaps in depression between low asset and high asset groups [19].

Higher socioeconomic status as defined by income is associated with lower levels of depression; this was true before the COVID-19 pandemic [26], and remained true after the COVID-19 pandemic began [18]. While income alone is an important factor associated with poor mental health, a growing body of work suggests that financial assets beyond income, namely, liquid savings [27–30], may also serve an important role in protecting against poor mental health. Assets refer to the financial, social, and physical resources that can be exchanged for goods and services that support health [19, 30–32]; these goods and services can be used to change contexts and reduce stress—and, in turn, can lessen depression. Household savings refer to money that families have in cash, savings or checking accounts, bonds, certificates of deposits, stocks, and pension funds [33]. While some types of savings are extremely liquid (such as cash or money in savings accounts), other types of savings may require penalties to use (such as early withdrawal from retirement accounts); however, these savings are still more liquid than physical assets, which require sale to acquire funds to pay for other needs. Our team was interested in liquid savings rather than physical assets such as homes, which require significant action to sell for cash if needed to cover other expenses. In this paper we focus on the role of financial assets, specifically highlighting income and savings, which are more easily accessible than other forms of assets in times of largescale disruption.

While much work has been done on the role of income broadly in shaping mental health outcomes particularly after largescale events [34, 35], less work has studied the role of wealth (beyond income) in informing mental health. We know that the role of savings and income are separately associated with lower prevalence of depression [28, 29, 36]. We do not know whether income and savings are jointly associated with depression over time, particularly in the context of the COVID-19 pandemic. Savings in particular may provide additional mental health protection in the face of disruptions [37]. For example, when a person loses a job, they also lose that income source. Savings may provide an additional cushion to soften the blow of unexpected events beyond income [38].

Building on this extant literature, we suggest that there are four asset groups that can describe lived experience: people with both high income and high savings; people with both low income and low savings; people with low income but high savings; and people with high income but low savings. Understanding how these four asset groups reported depression over the course of the COVID-19 pandemic could illuminate the role that different financial assets play in shaping depression. This paper aimed to 1) measure the prevalence of probable depression in a nationally representative sample of U.S. adults in April-May 2022, 2) track the evolution of probable depression annually over three waves during the COVID-19 pandemic, and 3) assess patterns in probable depression across the four asset groups, enhancing understanding of how income and savings together may influence depression over time in the context of a large global event.

## Methods

### Sample

Participants include adults ages 18 years and older who completed at least two waves of the COVID-19 and Life Stressors Impact on Mental Health and Well-being study (CLIMB) [16, 19, 32, 39]. Wave 1 of the survey was administered March 31, 2020 ‐ April 13, 2020 (T1), Wave 2 was administered March 23, 2021 ‐ April 19, 2021 (T2), and Wave 3 was administered March 22, 2022 ‐ April 19, 2022 (T3). The survey was administered through the AmeriSpeak standing panel, which recruits participants through a multi-stage, probability-based sampling process using a sample frame that represents 97% of U.S. households [40]. Participants were given the cash equivalent of $3 for completing the survey. Survey weights were created to account for non-response, survey selection, and demographics that when applied made the sample representative of the U.S. More information on the CLIMB sample can be found in previous writing [16, 17, 32]. The final analytic sample for this paper included 1,271 participants. Data were accessed and analyzed from May 2022 through April 2023. This study was approved by the Institutional Review Board (IRB) at NORC at the University of Chicago. The IRB at the Boston University Medical Campus reviewed the research and made the determination that it was not human subjects research.

### Key covariates

#### Probable depression

Probable depression was defined as a binary variable using a score of 10 or higher on the Patient Health-Questionnaire-9 (PHQ-9) [41]. The PHQ-9 has been validated to have a sensitivity of 88% and specificity of 88% as a screener for depression relative to the gold standard of a clinical diagnosis [42]. Probable depression was assessed at T1, T2, and T3.

#### Four asset groups

The four asset groups were defined by membership along income and savings groupings at T1. The asset groups were “low-low” representing low income with low savings, “high-low” representing high income with low savings, “low-high” representing low income with high savings, and “high-high” representing high income with high savings. High income was defined by reporting $65,000 or more in household income, roughly the median distribution for the sample and consistent with estimates for national family income [43, 44]. High savings was defined by reporting $20,000 or more in family savings, also roughly the median distribution for the survey sample and for the country according to other national estimates [43, 44]. Savings was defined at the household level in response to the following question: “We will now ask about household savings. By savings we mean money in all types of accounts, including cash, savings, or checking accounts, stocks, bonds, mutual funds, retirement funds (such as pensions, IRAs, 401Ks, etc), and certificates of deposit. What category best represents how much money your household (including yourself) has in savings?”

#### Demographic characteristics

Gender was defined as a binary variable (male/female). Age was defined as a continuous variable (years) in regression models. Race and ethnicity group was defined as a mutually exclusive categorical variable: non-Hispanic Asian, non-Hispanic Black, Hispanic, non-Hispanic White, and other or multiple races. Characteristics of the four asset groups were provided for the following variables: marital status (yes/no), college degree or above (yes/no), living in an urban area (yes/no), employment status (working full-time, working part-time, unemployed and looking for work, retired, and other), health insurance (employer based, private, Medicaid, Medicare, other, and none), political affiliation (Democrat, Republican, Independent, None), positive COVID-19 diagnosis ever (yes/no), and having received at least one dose of the COVID-19 vaccine (yes/no).

### Analysis

First, we described the characteristics of the four asset groups by our key demographic variables (i.e., gender, age, and race and ethnicity) and by descriptive variables. We reported unweighted frequencies and weighted percentages. Second, we estimated the weighted prevalence and 95% Confidence Intervals (CI) of probable depression at T1, T2, and T3 respectively by the four asset groups. Third, we graphed the weighted prevalence of probable depression at T1, T2, and T3 by income and savings status. Paired t-tests were used to assess if changes in probable depression were significant between each time period. Fourth, we estimated the odds and 95% CI of probable depression by income and savings group status at T1, T2, or T3 adjusting for gender, race and ethnicity, and age. Fifth, we estimated the odds of reporting probable depression at any point in the study (across T1, T2, or T3) for the four asset groups using generalized estimating equations (GEE) to cluster at the individual level given repeated measurements over time. Using GEE, we also estimated the weighted average of probable depression across time for each of the four asset groups. GEE models controlled for gender, race and ethnicity, and age at T1. Sensitivity analyses were conducted to assess sensitivity of results to different cutoffs for income and savings thresholds. Survey weights for individuals who responded to T1 and either T2 or T3 data collection were used for all analyses. Analyses were run in STATA 16.1 (StataCorp, College Station, TX).

## Results

Table 1 shows the demographic characteristics of the four asset groups. 47.5% of the weighted sample was part of the “low income-low savings” asset group (n = 550). 26.8% of the weighted sample was part of the “high income-high savings” asset group (n = 350). 11.4% of the weighted sample was part of “high income-low savings” asset group (n = 157) and 14.3% of the weighted sample was part of “low income-high savings” asset group (n = 173). While the sample was evenly split between female and male persons, the low income-low savings group had the greatest percentage of female persons (58.9%) relative to 48.3% of the high income-low savings group, 44.2% of the low income-high savings group, and 42.7% of high income-high savings group (p<0.001). Participants in the low income-low savings and high income-low savings America were younger than the low income-high savings and the high income-high savings groups. Among the high income-high savings group, 71.6% of the weighted sample was non-Hispanic White; among the low income-low savings group, 55.9% of the weighted sample was non-Hispanic White. The greatest number and percentage of non-Hispanic Black and Hispanic persons was in the low income-low savings group, with 21.9% of the weighted sample identifying as Hispanic and 15.3% of the weighted sample identifying as non-Hispanic Black.

**Table 1:** Characteristics of the study sample (N = 1,271) and the four asset groups.

	Total		Low income-low savings		High income-low savings		Low income-high savings		High income- high savings		P-value
	n	%	n	%	n	%	n	%	n	%	
**Total**	1,271		550	47.5	157	11.4	173	14.3	350	26.8	
**Gender**											<0.001
Female	622	51.3	330	58.9	74	48.3	66	44.2	129	42.7	
Male	649	48.7	220	41.1	84	51.7	107	55.8	221	57.3	
**Age (categorical)**											
18–24	70	11.7	38	16.1	9	10.7	8	7.2	13	6.7	<0.001
25–34	343	18.3	192	22.9	50	18.3	25	12.9	71	13.1	
35–44	219	16.1	95	15.7	40	24.4	19	12.4	57	15.4	
45–54	204	15.3	86	16.0	28	18.8	17	6.6	66	17.3	
55–64	212	17.6	73	15.3	16	13.2	37	19.0	78	22.9	
65+	223	21.0	66	14.1	14	14.7	67	41.9	65	24.7	
**Race and ethnicity**											<0.001
Asian, non-Hispanic	31	2.8	3	0.6	6	4.4	4	2.1	16	6.5	
Black, non-Hispanic	115	11.6	73	15.3	12	10.9	11	10.7	15	6.0	
Hispanic	216	16.8	128	21.9	24	11.8	24	14.5	32	11.2	
Other or multiple races	65	5.7	34	6.4	12	7.8	5	3.7	12	4.7	
White, non-Hispanic	844	63.0	312	55.9	103	65.1	129	69.1	275	71.6	
**Describing the four asset groups**			**Low income-low savings**		**High income-low savings**		**Low income-high savings**		**High income-high savings**		
**Married**	637	47.0	196	33.8	103	61.1	76	40.2	244	67.8	<0.001
**College degree or above**	419	33.0	95	16.1	60	41.1	54	33.6	199	65.5	<0.001
**Live in an urban area**	1,060	83.4	431	76.9	140	85.7	143	83.4	312	90.9	<0.001
**Employment**											<0.001
Working full-time	557	38.7	184	29.7	96	53.4	46	24.5	215	55.7	
Working part-time	135	10.7	70	12.8	20	12.8	12	8.0	29	8.8	
Unemployed and looking for work	114	9.3	75	12.9	9	6.3	14	7.4	15	5.4	
Retired	235	21.2	63	13.7	14	16.3	80	47.1	66	22.7	
Other	227	20.2	157	31.7	18	11.3	21	13.2	24	7.4	
**Health insurance**											<0.001
Employer-based insurance	494	35.5	132	22.3	91	52.6	43	24.5	217	57.3	
Private	158	12.1	53	9.6	23	13.7	28	16.1	48	13.7	
Medicare	289	27.8	125	28.1	18	17.3	72	44.8	61	22.7	
Medicaid	140	10.6	115	18.7	2	1.4	14	8.3	4	1.6	
Other	75	5.9	45	8.0	9	6.9	9	3.9	10	2.7	
None	110	8.1	78	13.3	14	8.1	6	2.4	10	2.0	
**Political affiliation**											<0.001
Democrat	437	36.6	185	33.9	53	36.2	62	39.0	124	39.9	
Republican	291	23.9	111	21.7	38	24.4	43	24.3	93	27.2	
Independent	313	25.2	119	23.1	38	25.2	47	25.9	95	28.5	
None	147	14.4	93	21.2	17	14.2	13	10.8	20	4.4	
**COVID-19 diagnosis**	143	12.0	70	14.5	13	9.2	16	7.9	38	11.0	0.215
**COVID-19 vaccine**	512	43.8	154	30.0	60	40.8	93	57.8	189	61.5	<0.001

Note: The four asset groups: “low-low” represents low income with low savings, “high-low” represents high income with low savings, “low-high” represents low income with high savings, and “high-high” represents high income with high savings. Low income was defined as below $65,000 and low savings was defined as below $20,000. Frequencies unweighted. Percentages weighted using survey weights for participants who participated in T1 and either T2 or T3 data collection. 41 of the 1,271 participants had missing income or savings data and are not reflected in the four asset groups. Values for specific characteristics (e.g., employment) do not add up due to missing values. Sample defined by participants who responded to at least 2 waves. Demographics at T1 used except for political affiliation, COVID-19 diagnosis, and COVID-19 vaccination, which are reported from T2.

Table 1 also provides additional descriptions of the four asset groups. Over two-thirds of the high income-high savings group was married (67.8%) relative to one-third (33.8%) of the low income-low savings group (p<0.001). 65.5% of the high income-high savings group had a college degree or more compared to 16.1% of the low income-low savings group (p<0.001). Over ninety percent of the high income-high savings group (90.9%) lived in an urban area while 76.9% of the low income-low savings group lived in an urban area. Over half (55.7%) of the high income-high savings group and less than a third (29.7%) of the low income-low savings group was working full-time. The highest distribution of persons working full-time was in the high income-high savings group, with 55.7% working full-time. Almost half of the low income-low savings group was on either Medicare (28.1%) or Medicaid (18.7%) relative to less than one-quarter of the high income-high savings group (Medicare: 22.7%; Medicaid: 1.6%) (p<0.001). The greatest distribution of Medicare participants was in the low income-high savings group, with 44.8% of the low income-high savings group using Medicare as their health insurance. 21.2% of the low income-low savings group did not identify with a political party compared to 4.4% of high income-high savings group (p<0.001). The high income-high savings group was more likely to have received at least one shot of the COVID-19 vaccine (61.5%) than the low income-low savings group (30.0%) (p<0.001). There was no significant difference in likelihood of having been diagnosed with COVID-19 across the four asset groups.

Table 2 shows the weighted prevalence of probable depression at T1, T2, and T3 nationally and across the four asset groups. The prevalence of probable depression at the national level increased from 26.5% (95% CI: 23.5, 29.7) at T1 to 31.6% (95% CI: 28.3, 35.2) at T2 and returned to 27.9% (95% CI: 24.4, 30.6) at T3. The low income-low savings group reported a prevalence of depression of 38.2% at T1, 47.7% at T2, and 42.2% at T3. The high income-low savings group reported a prevalence of probable depression of 18.2% at T1, 25.4% at T2, and 29.3% at T3. The low income-high savings group reported a prevalence of probable depression of 21.7% at T1, 21.8% at T2, 13.7% at T3. The high income-high savings group reported a prevalence of probable depression of 13.3% at T1, 13.8% at T2, and 12.5% at T3.

**Table 2:** Weighted prevalence of probable depression in 2020, 2021, and 2022 by four asset groupings.

	2020				2021				2022			
	n	%	95%	CI	n	%	95%	CI	n	%	95%	CI
**Overall**	321	26.5	(23.5,	29.7)	346	31.6	(28.3,	35.2)	290	27.9	(24.4,	30.6)
**Four asset groups**												
Low income- low savings	202	38.2	(33.0,	43.8)	224	47.7	(42.0,	53.6)	184	42.2	(36.3,	48.4)
High income-low savings	32	18.2	(12.4,	26.0)	38	25.4	(17.6,	35.1)	40	29.3	(20.3,	40.1)
Low income-high savings	36	21.7	(15.0,	30.4)	33	21.8	(15.1,	30.5)	26	13.7	(8.5,	21.4)
High income-high savings	42	13.3	(9.5,	18.4)	42	13.8	(10.0,	18.9)	36	12.5	(10.9,	16.0)

Note: T1: April ‐ May 2020; T2: April ‐ May 2021; T3: April ‐ May 2022. The four asset groups: “low-low” represents low income with low savings, “high-low” represents high income with low savings, “low-high” represents low income with high savings, and “high-high” represents high income with high savings. Low income was defined as below $65,000 and low savings was defined as below $20,000. Percentages weighted using survey weights for participants who participated in T1 and either T2 or T3 data collection. 41 of the 1,271 participants had missing income or savings data and are not reflected in the four asset groups. Probable depression defined by Patient Health Questionnaire-9 (PHQ-9) score ≥10.

**Fig 1:**
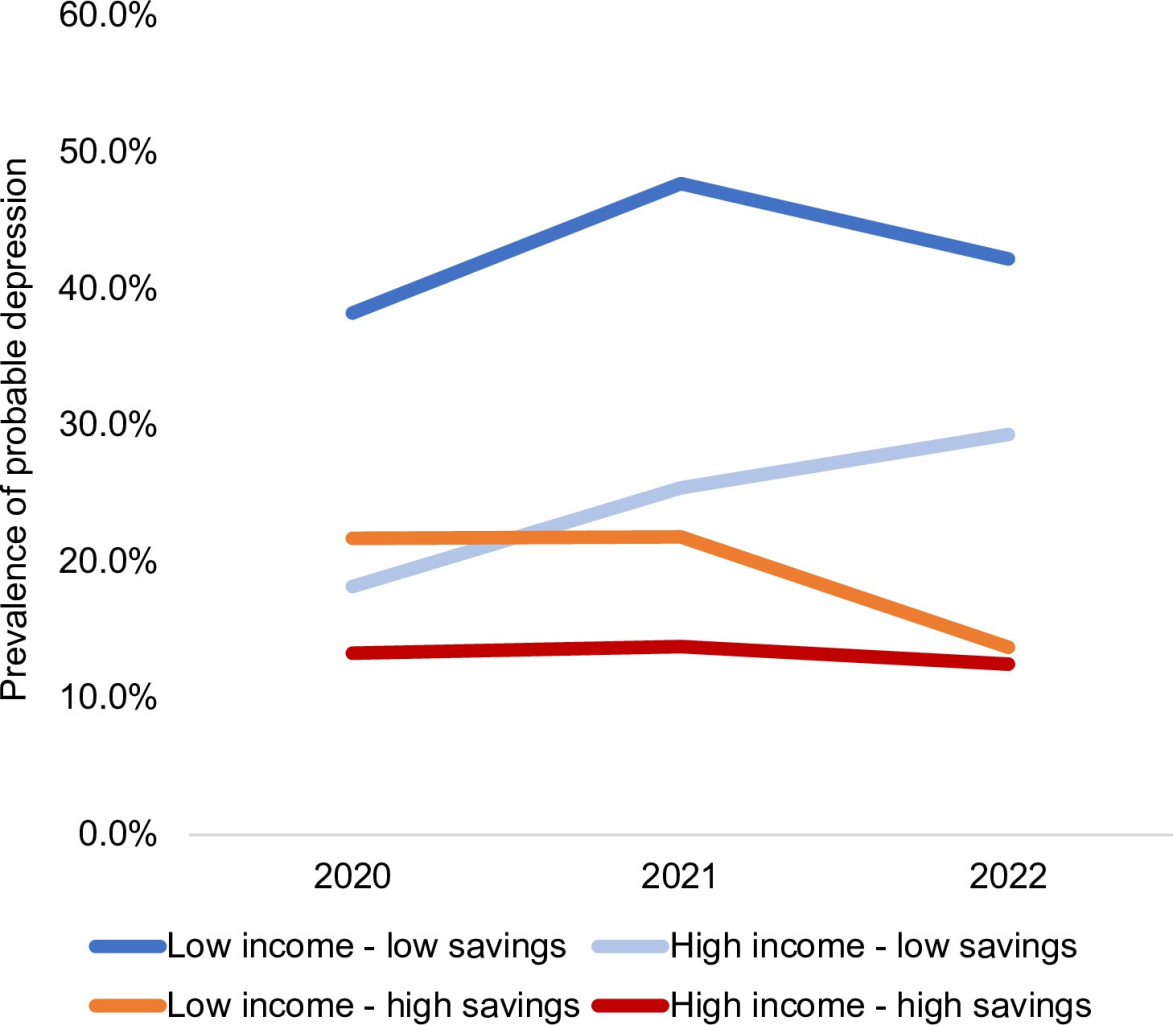
Weighted prevalence of probable depression by four asset groupings (income and savings) in 2020, 2021, and 2022. T1: Collected April ‐ May 2020; T2: Collected April ‐ May 2021; T3: Collected April ‐ May 2022. Low income was defined as below $65,000 and low savings was defined as below $20,000, based on median distribution in the sample. Percentages weighted using survey weights for participants who participated in T1 and either T2 or T3 data collection. 41 of the 1,271 participants had missing income or savings data and are not reflected in the four income and asset groups. Probable depression defined by Patient Health Questionnaire-9 (PHQ-9) score ≥10.

Fig 1 shows the prevalence of probable depression at each time (T1, T2, and T3) by four asset groupings. The low income-low savings group reported the highest level of probable depression across the three times, with a peak at T2. The high income-high savings group reported the lowest level of probable depression across the three times. The increase in probable depression from T1 to T2 was significant for the low income-low savings group. The increase in probable depression from T1 to T3 was statistically significant for the high income-low savings group. P-values for paired t-tests in changes over time for each asset group shown in S1 Table of S1 File.

Table 3 shows the weighted, adjusted odds of probable depression across the four asset groups adjusting for gender, race and ethnicity, and age. The low income-low savings group had 3.3 (95% CI: 2.1, 5.3) times the odds in T1, 5.0 (95% CI: 3.1, 7.9) times the odds in T2, and 3.7 (95% CI: 2.2, 6.0) times the odds of probable depression in T3 as the high income-high savings group. The high income-low savings group had 2.2 (95% CI: 1.2, 4.1) times the odds of probable depression in T3 as the high income-high savings group. The low income-high savings group also had 2.0 times the odds in T1 (95% CI: 1.1, 3.6) and 1.9 times the odds (95% CI: 1.1, 3.6) in T2, relative to the high income-high savings group.

**Table 3:** Multivariable logistic regression showing association between the four asset groups and probable depression in 2020, 2021, and 2022 controlling for age, gender, and race and ethnicity.

	2020			2021			2022		
	aOR	95% CI	P-value	aOR	95% CI	P-value	aOR	95% CI	P-value
**Four asset groups**									
Low income-low savings	3.3	(2.1, 5.3)	<0.001	5.0	(3.1, 7.9)	<0.001	3.7	(2.2, 6.0)	<0.001
High income-low savings	1.2	(0.7, 2.3)	0.492	1.8	(1.0, 3.2)	0.057	2.2	(1.2, 4.1)	<0.05
Low income-high savings	2.0	(1.1, 3.6)	<0.05	1.9	(1.1, 3.6)	<0.05	1.2	(0.6, 2.5)	0.552
High income-high savings	Ref	-		Ref	-		Ref	-	
**Gender**									
Female	1.7	(1.2, 2.5)	<0.01	1.3	(0.9, 1.8)	0.180	1.4	(1.0, 2.1)	0.057
Male	Ref	-		Ref	-		Ref	-	
**Age (years)**	1.0	(1.0, 1.0)	<0.01	1.0	(1.0, 1.0)	<0.001	1.0	(1.0, 1.0)	<0.001
**Race and ethnicity**									
Asian, non-Hispanic	0.9	(0.3, 3.4)	0.907	0.4	(0.1, 1.4)	0.151	0.3	(0.1, 1.2)	0.094
Black, non-Hispanic	0.8	(0.4, 1.5)	0.468	0.5	(0.3, 0.9)	<0.05	1.0	(0.5, 1.8)	0.873
Hispanic	1.0	(0.6, 1.7)	0.943	0.7	(0.4, 1.2)	0.229	1.1	(0.6, 1.8)	0.778
Other or multiple races	1.3	(0.6, 2.6)	0.501	1.4	(0.7, 2.8)	0.407	1.0	(0.4, 2.1)	0.937
White, non-Hispanic	Ref	-		Ref	-		Ref	-	

Note: T1: April ‐ May 2020; T2: April ‐ May 2021; T3: April ‐ May 2022. Four asset groups: Low income was defined as below $65,000 and low savings was defined as below $20,000. T1 used T1 demographics, T2 used T2 demographics, and T3 used T3 demographics. Weights for individuals who responded to T1 and either T2 or T3 data collection were used for all models. 41 of the 1,271 participants had missing income or savings data and are not reflected in the four asset groups. Probable depression defined by PHQ-9 score ≥10.

Table 4 shows the odds of having probable depression at any given timepoint, accounting for repeated measures for individuals and adjusting for gender, race and ethnicity, and age. The low income-low savings group had 3.7 (95% CI: 2.6, 5.3) times the odds, the high income-low savings group had 1.6 (95% CI: 1.0, 2.5) times the odds, and the low income-high savings group had 1.7 (95% CI: 1.0, 2.8) times the odds of reporting probable depression at any given timepoint compared to the high income-high savings group. Being female was associated with 1.4 (95% CI: 1.1, 1.9) times the odds of probable depression at any given timepoint relative to being male. The odds of probable depression at any time were not significantly different across race and ethnicity groups.

**Table 4:** Relation between four asset groups, demographic characteristics, and probable depression at any time during COVID-19 (2020, 2021, or 2022), adjusting for gender, age, race, and ethnicity.

	aOR	95%	CI	P-value
**Four asset groups**				
Low income-low savings	3.7	(2.6,	5.3)	<0.001
High income-low savings	1.6	(1.0,	2.5)	< .05
Low income-high savings	1.7	(1.0,	2.8)	.055
High income-high savings	Ref	-	-	-
**Gender**				
Female	1.4	(1.1,	1.9)	< .01
Male	Ref	-	-	-
**Age**	1.0	(1.0,	1.0)	<0.001
**Race and ethnicity**				
Asian, non-Hispanic	0.6	(0.2,	1.3)	0.714
Black, non-Hispanic	0.7	(0.5,	1.2)	0.082
Hispanic	1.0	(0.7,	1.4)	0.599
Other or multiple races	1.2	(0.7,	2.1)	0.108
White, non-Hispanic	Ref	-	-	-

Note: Generalized estimating equation (GEE) used to account for repeated measurements with assumption of exchangeable correlation. Four asset groups: Low income was defined as below $65,000 and low savings was defined as below $20,000. Probable depression defined by PHQ-9 score ≥10. Weights for individuals who responded to T1 and either T2 or T3 data collection were used. 41 of the 1,271 participants had missing income or savings data and are not reflected in the four asset groups.

**Fig 2:**
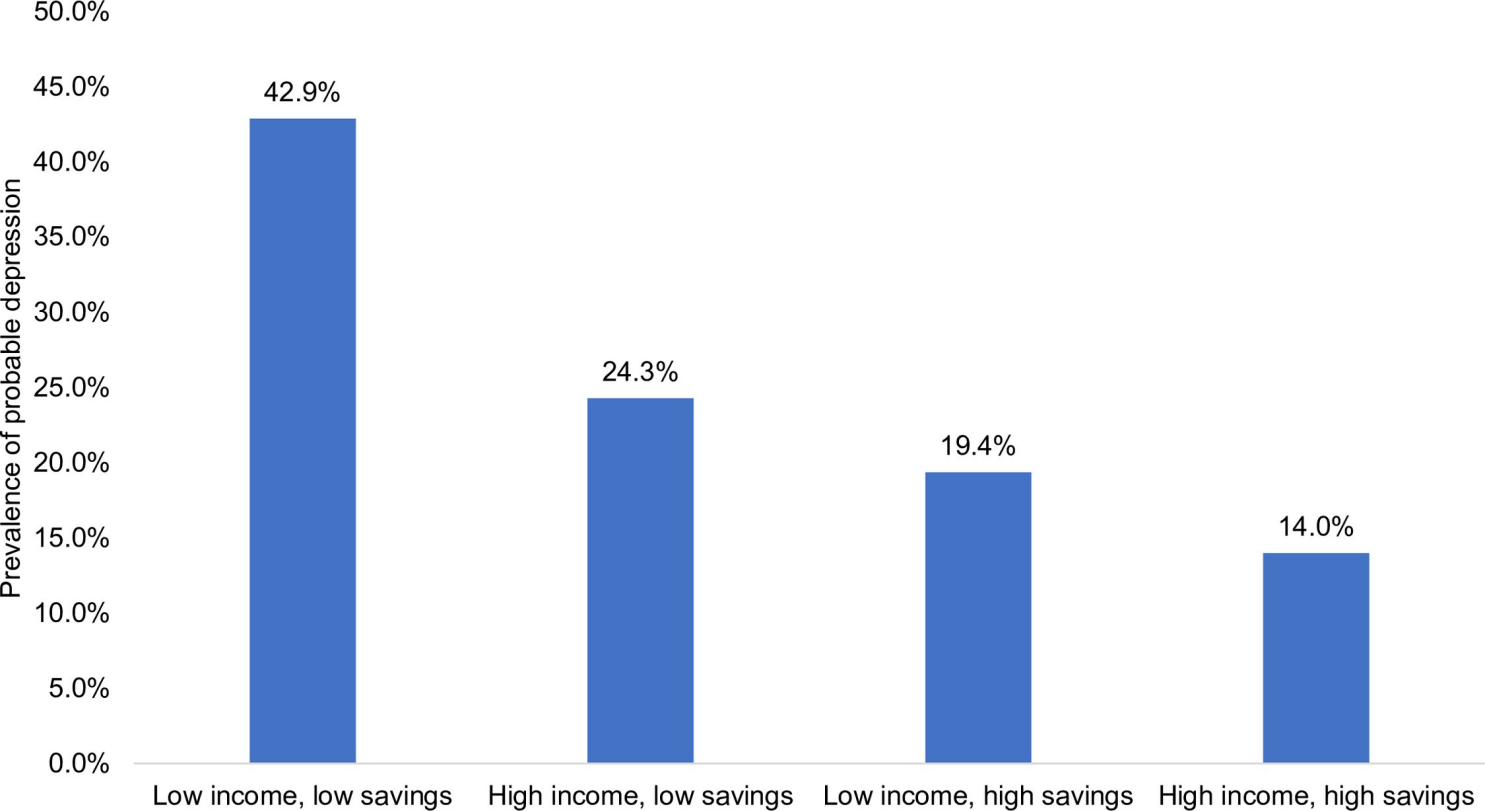
Average prevalence of probable depression across 2020, 2021, and 2022 by four asset groups. Weighted average of depression across time (2020, 2021, 2022) by four asset groups, accounting for repeated measures across individuals using generalized estimating equations (GEE). The four asset groups: “low-low” represents low income with low savings, “high-low” represents high income with low savings, “low-high” represents low income with high savings, and “high-high” represents high income with high savings. Low income was defined as below $65,000 and low savings was defined as below $20,000. Weights for individuals who responded to T1 and either T2 or T3 data collection were used. 41 of the 1,271 participants had missing income or savings data and are not reflected in the four asset groups. Probable depression defined by PHQ-9 score ≥10.

Fig 2 shows the average prevalence of probable depression across T1, T2, and T3 by four asset groups. 42.9% of the low income-low savings group reported probable depression while only 14.0% of the high income-high savings group reported probable depression across time. Approximately one quarter of the high income-low savings group reported probable depression and almost one fifth of the low income-high savings group reported probable depression.

S2–S5 Tables in S1 File present findings using different cutoffs for income and savings. Across different income and savings thresholds for asset groups, low income-low savings groups consistently reported higher odds of probable depression than high income-high savings groups.

## Discussion

Using a nationally representative, longitudinal panel study conducted annually over three time points during the first two years of the COVID-19 pandemic, we found that mental health experiences differed by savings and income asset groupings during the COVID-19 pandemic. Our three main findings were: 1) High probable depression previously documented at T1 (2020) and T2 (2021) remained high in T3 (2022). 2) Income and savings together may describe different dimensions of the social-ecological context that shapes probable depression over traditional measures of socioeconomic status such as income alone. 3) There was a substantial difference in the experience of probable depression over time as defined by financial assets groupings.

The continued high level of probable depression nationally is consistent with other studies that have measured mental health over the course of the pandemic. Household Pulse Survey (HPS) data reported by the CDC, showed a prevalence of 22.0 (95% CI: 21.2, 22.8) from April 27 –May 8, 2022 using the abbreviated PHQ-2 [45]. While this prevalence is slightly lower than our estimate of 26.8%, the estimate is still significantly higher than national estimates before the COVID-19 pandemic started [15, 16]. Additionally, the pattern of slight improvement in depressive symptoms for most groups documented here tracks similar patterns found in the HPS. Our work showed an increase in probable depression in 2021 followed by slight decrease (while still elevated compared to pre-pandemic times) in probable depression in 2022, consistent with findings from other studies that have monitored changes in mental health over the course of the pandemic. Haomiao et al. reported an increase in probable depression in U.S. adults on average from August to December 2020 and a reduction in probable depression on average from December 2020 to June 2021 [46]. Data collected earlier in the pandemic (2020) showed that improvements in mental health over the course of the pandemic were mediated by reductions in perceptions of risk around COVID-19 followed by changes in perceived financial strain due to COVID-19 and changes in lifestyle [47]. Some studies have shown improved depression trajectories relative to mental health at the start of the COVID-19 pandemic [48–50], but many of these studies were conducted internationally and ended in summertime, which may have imbued seasonal effects. Numerous studies have shown an increase in depression following the start of the COVID-19 pandemic [3, 50]; our work is consistent with the literature showing that depression remains elevated and similar to levels reported at the start of the pandemic [46, 51].

Our second finding that there were four distinct experiences of the COVID-19 pandemic based on savings and income profiles is consistent with an emerging literature on the importance of wealth in shaping depression [28]. For example, in a representative sample of older U.S. adults sampled before COVID-19, Gallo et al. found that low wealth individuals had worsened depressive symptoms several years after unexpected job loss whereas high wealth individuals did not show worsened depressive symptoms [37]. In a nationally representative sample of adults ages 18 and over interviewed before COVID-19, we previously showed that low savings was associated with probable depression using National Health and Nutrition Examination Survey (NHANES) data [29]. Using data collected in 2015–2016, we found that persons with less than $20,000 in savings had 1.5 times the odds of probable depression relative to persons with more, and that lower savings were associated with higher probable depression at every income level [29]. In a serial cross-sectional study of low-income U.S. adults collected from 2007 to 2016, we found that having less than $5,000 in savings was associated with 2.3 times the odds of major depressive disorder (MDD) [30]. In a scoping review of 96 articles published through July 2020, 58% of articles reported a significant inverse relation between wealth and depression [28]. The CLIMB study estimates of income and savings groupings were fairly consistent with distributions of assets across demographic groups in other national estimates [44].

This study was conducted in the U.S., and while these findings are likely not unique to the American context, this paper adds to the literature on how the American health experience differs across asset groups. Others have explored the notion that there are different lived experiences in the U.S. In 2006, Christopher Murray and colleagues reported on mortality differences across “Eight Americas” that were defined by race and counties [52]. Journalist George Packer defines “Four Americas” based on political leanings, philosophical approach, and educational attainment: Free America, Smart America, Real America, and Just America [53]. Economist Ellora Derenoncourt and colleagues report on the “Wealth of two nations” where they chart the trajectory of asset accumulation in Black versus White persons from 1860 to 2020 [54]. These three takes on fractured Americas all intersect with the accumulation of assets and ability of individuals to access economic opportunity. We found several key demographic differences in savings and income groupings: women and non-Hispanic Black persons were more likely to be in low-low America; older people were more likely to be in low-high America, and men and non-Hispanic white persons were more likely to be in high-high America. These findings are consistent with a literature that shows differences in wealth accumulation by race and ethnicity [54] and by gender [55]. We propose a new conception of groupings relevant for health in the US that takes into account not only income but also wealth [31]; understanding differences in wealth can potentially be an upstream source of understanding other differences in lived experiences across people in the U.S. before and exacerbated by the COVID-19 pandemic [56].

Our third finding documented differences in trajectories of depression over the course of the COVID-19 pandemic by income and savings grouping. Within the financial asset groupings, the low income-low savings group reported the highest levels of probable depression at every time point and the high income-high savings group reported the lowest levels of probable depression at every time point. These findings were consistent with the literature that shows that socio-economic indicators were associated with worse mental health during the COVID-19 pandemic [18, 19, 32, 57]. In an analysis of Household Pulse data from April 2020 to May 2021, Lee and Singh found that persons with income less than $25,000 had 2.3 times the odds of serious depression compared to persons with income of more than $200,000 [20]. They reported that adults with a household income of $25,000 or less had a weighted prevalence of 20.45% for serious depression relative to 5.19% for persons with an income of $200,000 or more. A study conducted in Australia found that higher income was associated with worse mental health outcomes in response to lockdowns [58]; however, this may be due to a higher proportion of high income individuals living in more urbanized areas, which had greater surveillance during the COVID-19 lockdowns. Our findings are consistent with findings from other countries that explore constructs of savings or wealth—or financial strain—beyond income. For example, in a longitudinal study of adults in the Netherlands over the first year of the pandemic, having lower savings leading into the pandemic predicted financial strain during the pandemic which preceded worse mental health [59]. One potential explanation for the association between wealth and depression is the ability to control one’s context; a study in Hong Kong showed that persons with more assets reported fewer disruptions to primary and secondary routines during the COVID-19 pandemic, less financial stress, and less persistent depression than their counterparts with fewer assets [60]. Outside of the COVID-19 pandemic, different types of liquid financial assets have been shown to be associated with mental health outcomes in the U.S. and in Europe [61].

### Limitations

This study has three principal limitations. First, the PHQ-9 is a depression screener and is not a clinical diagnosis of depression by a health provider; however, the screener has been validated to have 88% sensitivity and 88% specificity relative to the gold standard of a medical provider diagnosis [42]. The language of probable depression has been used in other large national studies to identify that the PHQ-9 is a screener [41]. Others have suggested that the PHQ-9 can be used in conjunction with structured interviews for a formal depression diagnosis [62, 63]. Additionally, this study explored a binary definition of probable depression, which has clinical significance. It is possible that depression severity trajectories varied across the pandemic. This study focused on exceeding a clinically meaningful PHQ-9 cutoff, with clear action for practitioners and patients consistent with the U.S. Preventive Services Task Force on Depression and Suicide Risk Screening in Adults [64]. Second, other assets such as home ownership and educational attainment may influence depression during COVID-19; however, this paper was designed to look at income and savings in particular, given the flexibility that liquid assets (such as cash) can provide in meeting expenses as opposed to illiquid assets such as educational status or home ownership, which cannot be used directly to cover expenses. Third, it is possible that the relation between assets and mental health varied across states, potentially due in part to differences in both COVID-19 infection, state generosity of social and financial benefits, and cost of living across states. The study was not designed to explore state variation, and exploring heterogeneity in the relation of assets to mental health across states would be worthy of investigation in the future.

## Conclusion

Notwithstanding these limitations, these findings suggest that probable depression stayed relatively stable and high from 2020 through 2022. There were different trajectories experienced by persons across the four asset groups, with persons in the low income-low savings group experiencing the highest levels of probable depression relative to all other groups. High income-low savings groups in particular reported higher levels of probable depression two years into the pandemic relative to the start of the pandemic. The group experiencing the lowest levels of probable depression during the COVID-19 pandemic was the high-income-high savings group. It is possible that efforts to improve the mental health of low income-low savings America could start with improving either income or savings and that optimal mental health could be achieved when persons have both adequate income and savings. Policies that improve the financial stability and savings of lower income groups may improve mental health. The four asset groups experienced different mental health trajectories; future efforts to improve population health may wish to acknowledge and address the differential experiences of these groups and the upstream factors leading to disparities in depression so that the country may achieve maximal mental health.

## Supporting information

S1 File Supporting information file for study. (PDF)

S1 Data Study data in .csv format. (CSV)
