# Supplementary material for: Depression and assets during the COVID-19 pandemic: A longitudinal study of mental health across income and savings groups
Source: PLoS One. 2024 Jun 14;19(6):e0304549. doi: 10.1371/journal.pone.0304549 (PMC11178170; doi:10.1371/journal.pone.0304549)
Supplement: S1 File — (PDF) [file pone.0304549.s001.pdf]

Supporting information

S1 Table. Changes in probable depression over time

Four asset groups	T1 to T2		T2 to T3		T1 to T3	
	Mean difference	p-value	Mean difference	p-value	Mean difference	p-value
Low income-low savings	0.07	**<0.01**	-0.03	0.225	0.02	0.317
High income-low savings	0.05	0.127	0.02	0.533	0.10	**<0.05**
Low income-high savings	0.00	1.000	-0.05	0.109	-0.03	0.372
High income-high savings	0.02	0.412	-0.01	0.706	0.01	0.506

Note: CLIMB data used. Paired t-test conducted to generate mean differences over time and p-values. T1: April - May 2020; T2: April - May 2021; T3: April - May 2022. Probable depression defined by PHQ-9 score ≥10. Unweighted. 41 of the 1,271 participants had missing income or savings data and are not reflected in the four asset groups.

S2 Table. Multivariable logistic regression showing association between the four asset groups and probable depression in 2020, 2021, and 2022 controlling for age, gender, and race and ethnicity using higher median income threshold (≥$75,000) and same savings threshold (≥$20,000) as the main manuscript

	Time 1 (2020)			Time 2 (2021)			Time 3 (2022)		
**Characteristic**	**OR[1]**	**95% CI[1]**	**p-value**	**OR[1]**	**95% CI[1]**	**p-value**	**OR[1]**	**95% CI[1]**	**p-value**
**Four asset groups**									
High income-high savings	—	—		—	—		—	—	
Low income-low savings	2.9	1.8, 4.6	**<0.001**	4.6	2.8, 7.4	**<0.001**	3.5	2.1, 5.9	**<0.001**
Low income-high savings	1.5	0.8, 2.7	0.182	1.6	0.9, 2.9	0.142	1.1	0.6, 2.1	0.787
High income-low savings	1.3	0.7, 2.5	0.453	1.5	0.7, 2.8	0.270	1.7	0.9, 3.5	0.113
**Female**	1.7	1.2, 2.4	**<0.01**	1.3	0.9, 1.8	0.193	1.4	1.0, 2.1	0.057
**Age**	1.0	1.0, 1.0	**<0.01**	1.0	1.0, 1.0	**<0.001**	1.0	1.0, 1.0	**<0.001**
**Race and ethnicity**									
White, non-Hispanic	—	—		—	—		—	—	
Black, non-Hispanic	0.8	0.4, 1.5	0.515	0.5	0.3, 0.9	**<0.05**	0.9	0.5, 1.8	0.843
Asian, non-Hispanic	0.9	0.3, 3.2	0.885	0.4	0.1, 1.5	0.172	0.4	0.1, 1.3	0.114
Other, non-Hispanic	1.3	0.6, 2.5	0.504	1.4	0.7, 2.8	0.393	1.0	0.5, 2.1	0.977
Hispanic	1.1	0.7, 1.7	0.804	0.8	0.5, 1.2	0.274	1.1	0.6, 1.8	0.759

[1] OR = Odds Ratio, CI = Confidence Interval

Note: CLIMB data used. Sensitivity analysis with higher threshold for median household income (≥$75,000 instead of $65,000 as in the main paper) and savings level unchanged. T1: April - May 2020; T2: April - May 2021; T3: April - May 2022. The four asset groups: "low-low" represents low income with low savings, "high-low" represents high income with low savings, "low-high" represents low income with high savings, and "high-high" represents high income with high savings. Weights for individuals who responded to T1 and either T2 or T3 data collection were used for all models. 41 of the 1,271 participants had missing income or savings data and are not reflected in the four asset groups. Probable depression defined by PHQ-9 score ≥10. Model controls for age, gender, and race and ethnicity.

S3 Table. Relation between four asset groups, demographic characteristics, and probable depression at any time during COVID-19 (2020, 2021, or 2022) using higher median income threshold (≥$75,000) and same savings threshold (≥$20,000) as the main manuscript

**Characteristic**	**OR**[1]	**95% CI**[1]	**p-value**
**Four asset groups**			
High income-high savings	—	—	
Low income-low savings	3.4	2.3, 4.9	**<0.001**
Low income-high savings	1.3	0.8, 2.2	0.241
High income-low savings	1.4	0.8, 2.4	0.193
**Female**	1.4	1.1, 1.9	**<0.01**
**Age**	1.0	1.0, 1.0	**<0.001**
**Race and ethnicity**			
White, non-Hispanic	—	—	
Black, non-Hispanic	0.8	0.5, 1.2	0.238
Asian, non-Hispanic	0.6	0.2, 1.4	0.209
Other, non-Hispanic	1.3	0.7, 2.1	0.387
Hispanic	1.0	0.7, 1.4	0.953

[1] OR = Odds Ratio, CI = Confidence Interval

Note: CLIMB data used. Sensitivity analysis with higher threshold for median household income (≥$75,000 instead of $65,000 as in the main paper) and higher savings level unchanged. Generalized estimating equation (GEE) used to account for repeated measurements with assumption of exchangeable correlation. Four asset groups: Low income was defined as below $75,000 and low savings was defined as below $20,000. Probable depression defined by PHQ-9 score ≥10. 41 of the 1,271 participants had missing income or savings data and are not reflected in the four asset groups. Weights for individuals who responded to T1 and either T2 or T3 data collection were used.

S4 Table. Multivariable logistic regression showing association between the four asset groups and probable depression in 2020, 2021, and 2022 controlling for age, gender, and race and ethnicity using higher median income threshold (≥$75,000) and higher savings threshold (≥$25,000) than main manuscript

	**Time 1**			**Time 2**			**Time 3**		
**Characteristic**	**OR**[1]	**95% CI**[1]	**p-value**	**OR**[1]	**95% CI**[1]	**p-value**	**OR**[1]	**95% CI**[1]	**p-value**
**Four asset groups**									
High income-high savings	—	—		—	—		—	—	
Low income-low savings	4.8	2.9, 7.9	**<0.001**	3.5	2.0, 6.0	**<0.001**	3.4	2.3, 4.9	**<0.001**
Low income-high savings	1.6	0.9, 3.0	0.123	1.0	0.5, 2.0	0.995	1.3	0.8, 2.2	<0.241
High income-low savings	1.7	0.9, 3.	0.100	1.7	0.9, 3.5	0.109	1.4	0.8, 2.4	<0.193
**Female**	1.3	0.9, 1.8	0.160	1.5	1.0, 2.2	**<0.05**	1.4	1.1, 1.9	**<0.01**
**Age**	1.0	1.0, 1.0	**<0.001**	1.0	1.0, 1.0	**<0.001**	1.0	1.0, 1.0	**<0.001**
**Race and ethnicity**									
White, non-Hispanic	—	—		—	—		—	—	
Black, non-Hispanic	0.5	0.3, 0.9	**<0.05**	0.9	0.5, 1.7	0.801	0.8	0.5, 1.2	0.238
Asian, non-Hispanic	0.4	0.1, 1.5	0.183	0.4	0.1, 1.3	0.114	0.6	0.2, 1.4	0.209
Other, non-Hispanic	1.4	0.7, 2.8	0.367	1.0	0.5, 2.2	0.969	1.3	0.7, 2.1	0.387
Hispanic	0.7	0.5, 1.2	0.245	1.1	0.6, 1.8	0.858	1.0	0.7, 1.4	0.953

[1] OR = Odds Ratio, CI = Confidence Interval

Note: CLIMB data used. Sensitivity analysis with higher threshold for median household income (≥$75,000 instead of $65,000 as in the main paper) and higher savings (≥$25,000) instead of $20,000. T1: April - May 2020; T2: April - May 2021; T3: April - May 2022. The four asset groups: "low-low" represents low income with low savings, "high-low" represents high income with low savings, "low-high" represents low income with high savings, and "high-high" represents high income with high savings. Weights for individuals who responded to T1 and either T2 or T3 data

collection were used for all models. 41 of the 1,271 participants had missing income or savings data and are not reflected in the four asset groups. Probable depression defined by PHQ-9 score ≥10. Model controls for age, gender, and race and ethnicity.

Supplemental Table 5. Relation between four asset groups, demographic characteristics, and probable depression at any time during COVID-19 (2020, 2021, or 2022) using higher median income threshold (≥$75,000) and higher savings threshold (≥$25,000) than main manuscript

Characteristic	OR[1]	95% CI[1]	p-value
**Four asset groups**			
High income-high savings	—	—	
Low income-low savings	3.3	2.3, 4.9	**<0.001**
Low income-high savings	1.3	0.8, 2.1	0.178
High income-low savings	1.4	0.9, 2.4	0.323
**Female**	1.5	1.1, 1.9	**<0.01**
**Age**	1.0	1.0, 1.0	**<0.001**
**Race and ethnicity**			
White, non-Hispanic	—	—	
Black, non-Hispanic	0.8	0.5, 1.2	0.214
Asian, non-Hispanic	0.6	0.2, 1.4	0.209
Other, non-Hispanic	1.3	0.8, 2.2	0.336
Hispanic	1.0	0.7, 1.4	0.888

[1] OR = Odds Ratio, CI = Confidence Interval

Note: CLIMB data used. Sensitivity analysis with higher threshold for median household income (≥$75,000 instead of $65,000 as in the main paper) and higher savings level (≥$25,000 instead of $20,000 as in the main paper). Generalized estimating equation (GEE) used to account for repeated measurements with assumption of exchangeable correlation. Four asset groups: Low income was defined as below $75,000 and low savings was defined as below $20,000. Probable depression defined by PHQ-9 score ≥10. 41 of the 1,271 participants had missing income or savings data and are not reflected in the four asset groups. Weights for individuals who responded to T1 and either T2 or T3 data collection were used.
